# The possible role of long non-coding RNAs in recurrent miscarriage

**DOI:** 10.1007/s11033-022-07427-9

**Published:** 2022-04-10

**Authors:** Yanan Zhang, Shan Wang

**Affiliations:** 1 Shandong Key Laboratory of Reproductive Medicine, Department of Obstetrics and Gynecology, Shandong Provincial Hospital Affiliated to, Shandong First Medical University, 324 Jingwu Road, Jinan, 250021 China; 2 Department of Obstetrics and Gynecology, Shandong Provincial Hospital, Shandong University, 324 Jingwu Road, Jinan, 250021 China

**Keywords:** lncRNA, Recurrent miscarriage, Susceptibility, Endometrial receptivity, Maternal-foetal interface

## Abstract

Recurrent miscarriage (RM) is a complicated disease in reproductive medicine that impacts many families. Currently, the etiology of RM is thought to include chromosome abnormalities, reproductive tract malformations, autoimmune dysfunction, infection, and environmental factors. However, the underlying mechanisms of RM remain unknown. At present, research on long non-coding RNAs (lncRNAs) is rapidly emerging and becoming a hot research topic in epigenetic studies. Recent studies revealed that lncRNAs are strongly linked to RM and play a crucial role in epigenetic, cell cycle, cell differentiation regulation, and other life activities. This article mainly reviews the difference in lncRNA expression in patients with RM and regulation of susceptibility, endometrial receptivity, and the maternal-fetal interface. Meanwhile, the correlation between lncRNAs and RM is expounded, which provides new insights for the early diagnosis and treatment of RM.

## Introduction

Recurrent miscarriage (RM) refers to the pregnancy loss before 20 weeks of gestation or a fetal weight less than 500 g for two or more consecutive pregnancies [1]. Most RM occurs when they are clinically diagnosed as occurring in the pre-embryonic or embryonic stage [2]. Early pregnancy loss occurs in approximately 11% of pregnant women at 8–12 gestational weeks [3], and the incidence of RM is about 2% among all pregnancy outcomes [4].

RM is a significant clinical problem in reproductive health and affects family well-being, but unfortunately, more than half of RM patients are unable to identify the etiological factors [5]. The leading causes of RM are maternal chromosomal abnormalities, genital tract abnormalities, immune dysfunction, endocrine disorders, the presence of genital tract infection, and cervical insufficiency [6]. Although chromosomal abnormalities are currently considered the leading cause of RM, there are still most cases where the cause is not yet known.

RNA sequencing, gene expression profiling by microarray and transcriptome analysis increasingly indicate the role of non-coding RNAs (ncRNAs) as critical regulators of gene expression and signal transduction [7]. And yet, only 2% of the human transcribed genome is protein-coding genes. Long non-coding RNAs (lncRNAs) are a specific type of ncRNAs, which play significant regulatory roles in health and disease [8–11]. LncRNAs transcripts are to participate in epigenetic remodeling, subcellular localization and transcriptional regulation. Aberrantly regulated lncRNAs are implicated in cancer through downregulation or upregulation of the specific lncRNAs associated with the adjacent normal tissue [12]. Consequently, lncRNAs act similarly to tumor proto-oncogenes or suppressor genes.

Studies have confirmed that long non-coding RNAs are involved in RM and regulate embryonic development, endometrial receptivity, and embryo-maternal interactions by regulating gene expression. Consequently, it is imperative for RM to explore the underlying mechanisms and seek new treatments in order to reduce the harm to families.

## Overview of lncRNAs

LncRNAs are nucleic acid sequences that are lengths greater than 200 nt and do not encode proteins [13]. LncRNAs are mainly transcribed by RNA polymerase II and have an mRNA-like structure, typically with a 7mC cap at the 5′ end and a polyA tail at the 3′ end [14]. They account for approximately 27% of human-annotated genes and have a variety of mechanisms, which distinguish them from other small non-coding RNAs, such as miRNAs, piRNAs, siRNAs, and several others [15].

Accumulating evidence has indicated that lncRNA plays a critical role in biological processes. According to their genome location and background, lncRNAs can be divided into intronic lncRNAs, sense lncRNAs, antisense lncRNAs, intergenic lncRNAs, and bidirectional lncRNA [16]. The intronic lncRNA is derived from the intronic region of genes encoding proteins, originating from the antisense and sense regions of the intronic region, such as SPRY4-ITI and CHRF [17, 18]. The sense and antisense lncRNA are transcribed from the sense and antisense strands of the genome encoding protein genes, like ANRIL and COLDAIR [18, 19]. Intergenic lncRNA is known as large intervening non-coding RNA, transcribed from the intergenic region of genes encoding proteins, such as MALAT1, MIAT, and H19, etc. [17, 18]. Bidirectional lncRNA is originated from different directions of protein-coding genes, including HCCL5 and LEENE [19]. Although lncRNAs do not encode proteins, they play crucial roles in various biological processes. Firstly, lncRNAs exist in almost all living things, suggesting that they are primary components of organisms. LncRNAs account for a high proportion of RNAs in complex organisms, indicating that they serve a significant role in increasing complexity of eukaryotes [20]. Secondly, lncRNAs have high levels of tissue-specific expression, which is specifically manifested in the fact that the lncRNAs expression levels in diverse tissues are different, and distinct expression patterns are present in other parts of the same tissue [21]. Furthermore, lncRNAs have significant temporal and spatial specificity, and the expression of the same lncRNA varies significantly in different developmental stages of the same tissue or organ [22].

In early studies, due to the low level of transcription of lncRNAs, they were considered the “noise” of transcription [23]. However, with the improvement of experimental technology, the function of lncRNAs was found to be closely associated with their location. The use of FISH and other tools to determine the subcellular location of lncRNAs is conducive to studying their mechanism of action [24]. LncRNAs located in the nucleus can participate in gene regulation processes, including promoter-specific inhibition, transcription activation, and epigenetic regulation [25]. LncRNA regulates gene expression by directly binding RNA polymerase and transcription factors or by interfering with the binding of promoters and polymerase [26]. Moreover, lncRNAs regulate the chromatin structure through different functional steps, including histone modification, DNA methylation, and chromatin remodeling [27]. Additionally, lncRNAs located in the cytoplasm participate in post-transcriptional gene regulation, including the regulation of mRNA stability, miRNA translation, and signal transduction pathways [28]. LncRNAs facilitate the post-transcriptional processing of mRNA by recognizing complementary sequences in mRNA, such as splicing, transport from the nucleus to the cytoplasm, editing, etc., to obtain a mature form [29]. Meanwhile, lncRNAs act as a sponge, also called competitive endogenous RNAs (ceRNAs), containing sequences complementary to miRNA sequences, thereby isolating miRNA sequences and preventing them from binding to the target [29]. Moreover, lncRNAs may promote or inhibit translation by interacting with initiation factors, ribosomes, or ribosomal RNA [16]. The above findings suggest that lncRNAs can alter the stability of cells and tissues through these functions, which in turn can cause a variety of diseases. There is accumulating evidence for the critical role of lncRNAs in the tumourigenesis and progression of Hepatocellular carcinoma (HCC). A variety of HCC-associated lncRNAs have been proven to be aberrantly expressed and involved in cancer phenotypes (for example, sustained proliferation, evasion of apoptosis, accelerated angiogenesis and acquisition of invasive capacity) through binding to DNA, RNA or proteins or encoding small peptides [30]. Additionally, PCGEM1 introduces the Pygopus family PHD finger 2 into the enhancer-promoter region of the AR gene, modulates AR-induced gene expression, is overexpressed in prostate cancer, and promotes cell proliferation [31]. In breast cancer, lncRNA ANRIL induces gene silencing at the INK4b-ARF-INK4a locus by interacting with CBX7 (PRC1 component) and SUZ12 (PRC2 component) and regulates its adjacent tumor suppressor CDKN2A/B through epigenetic mechanisms, thereby controlling cell proliferation and senility [32]. The lncRNA CCAT1 acts as a competitive endogenous RNA (ceRNA) for miR-155 and inhibits c-Myc expression, which has been implicated in the pathogenesis of myeloid leukemia (AML), colorectal cancer, esophageal cancer and lung cancer [33].

## Differences of lncRNA expression in recurrent miscarriage

**Fig. 1:**
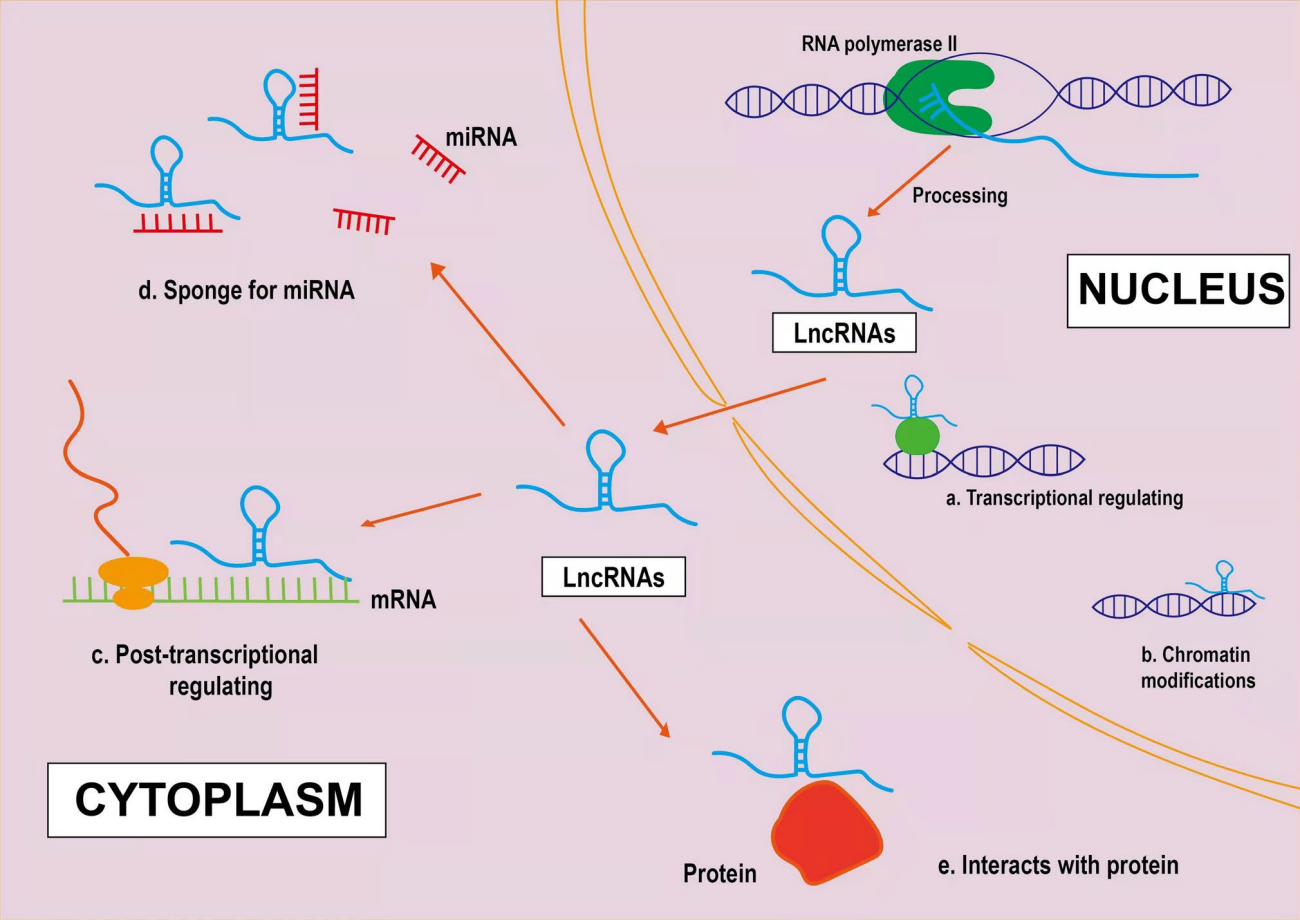
Biogenesis and biological roles of long non-coding RNAs (lncRNAs). LncRNAs are mainly transcribed by RNA polymerase II and have an mRNA-like structure, typically with a 7mC cap at the 5′ end and a polyA tail at the 3′ end. **a** Some lncRNAs promote or suppress gene expression at transcriptional levels. **b** Some lncRNAs regulate gene expression by assembling chromatin-modifying complexes. **c** lncRNAs located in the cytoplasm participate in posttranscriptional gene regulation. **d** lncRNAs can act as miRNA sponges. **e** lncRNAs interact with proteins

Several studies have demonstrated that lncRNAs are differentially expressed in RM and exhibit tissue-specific expression in embryo sacs (Table 1). A total of 4421 lncRNAs were quantitatively detected in early PCR experiments as having differential expression in chorionic villi, of which 1537 were upregulated, 2884 were downregulated [34]. Meanwhile, 6771 lncRNAs were differentially expressed in the maternal decidua, of these, 3154 lncRNAs were upregulated, and 3617 were downregulated, indicating that differential expression of lncRNAs is more significant in the decidua than in villi [34]. Moreover, Wang et al. [35] identified 1449 differentially expressed lncRNAs (467 upregulated lncRNAs and 982 downregulated lncRNAs) in chorionic villi of RM patients compared with healthy women. And, KEGG pathway analysis revealed that these upregulated and downregulated lncRNAs might target 26 pathways that correspond to transcripts, including 11 upregulated and 15 downregulated pathways [35]. The upregulated lncRNAs participate in the steroid hormone biosynthesis, fatty acid metabolism, glycerophospholipid metabolism, RNA polymerase, ecm receptor interaction process, and the downregulated lncRNAs participate in androgen and oestrogen metabolism, galactose metabolism, purine metabolism, RNA polymerase, glycosphingolipid biosynthesis, indicating that lncRNAs may participate in the pathogenesis of RM by affecting maternal endocrine homeostasis [35]. Furthermore, KEGG pathway analysis also demonstrated that differentially expressed lncRNAs participate in immune-related pathways, indicating that they may regulate the pathogenesis of RM. A critical pathway affected by the upregulation of lncRNAs is the extracellular matrix (ECM) receptor interaction, and GO analysis showed that most lncRNAs are involved in binding and molecular interactions [35]. The endocrine, immune, ECM receptor interaction and apoptosis pathways are the main mechanisms that participate in the pathogenesis of RM. Therefore, it is tempting to speculate that the differentially expressed lncRNAs affect cell adhesion through ECM receptor interactions in the placenta of patients with RM. However, only a few of the mechanisms of lncRNAs have been identified in current studies, and the underlying mechanisms of most other lncRNAs need to be explored (Fig. 1).

**Table 1:** Differences of lncRNA expression in RM

Sample	Method	Upregulated	Downregulated	Refs
Chorionic villi	Microarray qRT-PCR	1537 lncRNAs	2884 lncRNAs	[34]
Maternal decidua	Microarray qRT-PCR	3154 lncRNAs	3617 lncRNAs	[35]
Chorionic villi	Microarray qRT-PCR	467 lncRNAs	982 lncRNAs	[35]

*LncRNA* long non‑coding RNA, *RM* recurrent miscarriage

## The mechanism of lncRNAs in recurrent miscarriage

### Susceptibility

Susceptibility refers to the degree of susceptibility of humans or animals to infection by a particular pathogen [36], and the genetic material determines the individual's risk of disease. It can also be understood as the risk of different individuals being infected in the same environment. Susceptibility is genetically determined, and genes often play a more critical role under the influence of pathogenic environmental factors [36]. Numerous studies indicated that genetic variation in genes that regulate cellular biological behaviors may be linked to susceptibility to RM (Table 2).

**Table 2:** Underlying mechanisms of RM and their associated lncRNAs

Classification	Underlying mechanism	Associated lncRNA	Refs
1	Susceptibility	HULC,CCAT2,MALAT1	[37–42]
2	Endometrial receptivity	H19,TUNAR,CECR3, ST7-OT3, DHRS4-AS1, C22orf34, RAMP2-AS1, PNCT-HSA157732	[43–45]
3	The maternal-foetal interface: Cellular level	lnc-49a,ANRIL,SLC4A1-1,HOTAIR,HZ08,HZ01	[46–53]
4	The maternal-foetal interface: Organizational level	H19	[54, 55]

*lncRNA* long non‑coding RNA, *HULC* highly upregulated in liver cancer, *CCAT2* Colon cancer-associated transcript 2, *MALAT1* metastasis associated lung adenocarcinoma transcript-1, *TUNAR* TCL1 upstream neural differentiation-associated RNA, *HOTAIR* HOX antisense intergenic RNA

#### HULC

The HULC (highly up-regulated in liver cancer) gene is located on chromosome 6p24.3, approximately 500 nt in length, promoting different cell phenotypes, including proliferation, survival, and invasion in vivo [56, 57].

**Fig.2:**
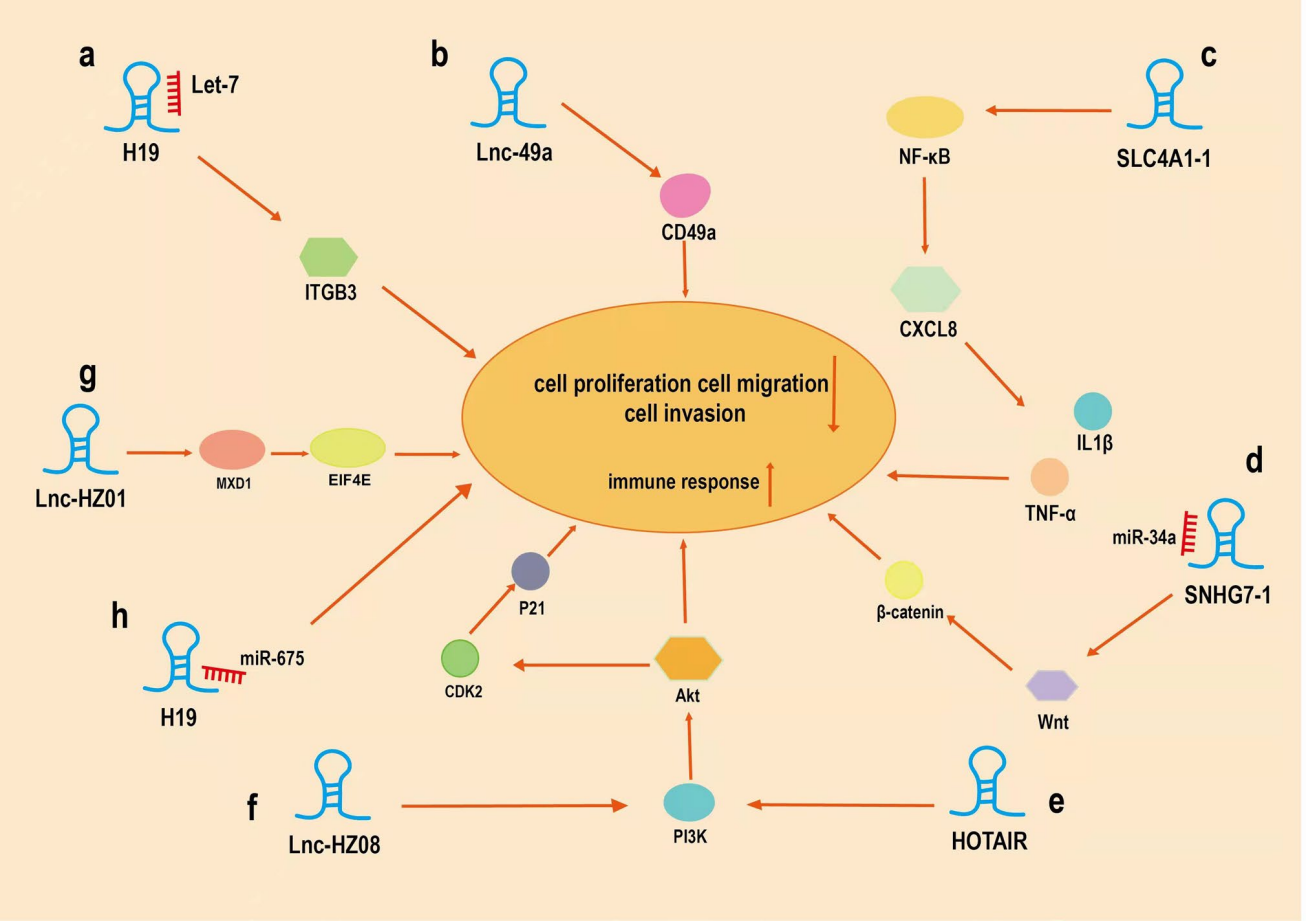
Underlying mechanisms of some lncRNAs in recurrent miscarriage (RM). Some lncRNAs play crucial roles in RM by sponging miRNAs or interacting with other proteins. **a** H19 interacts with miRNAs of Let-7 to regulate the transcription and translation of integrin β3. **b** lnc-49a can positively regulate CD49a expression. **c** lnc-SLC4A1-1 recruit NF-κB and bind to the CXCL8 promoter, which leads to upregulation of CXCL8. **d** lncRNA SNHG7 interacts with miR-34a to regulate the progression of RM through the Wnt/β-catenin signaling pathway. **e** lncRNA HOTAIR activates the PI3K-AKT signaling pathway to promote MMP-2 expression. **f** overexpression lnc-HZ08 suppressed the PI3K and AKT protein levels, and the downstream proteins CDK2 and p-P21. **g** lnc-HZ01 promotes the transcription of MXD1 mRNA, which promotes the transcription of METTL14 mRNA. **h** H19 interacts with miR675 to regulate the progression of RM.

Several studies demonstrated that susceptibility to diverse diseases is related to HULC genetic polymorphisms [58, 59]. Fang et al. [37] screened four SNPs in the HULC gene and determined that variant genotypes of rs1041279 C > G, rs17144343 G > A and rs7770772 G > C were linked to a reduced risk of RM, which demonstrated that the rs17144343 GA/AA allele, rs7770772 GC/CC alleles, rs1041279 GG alleles of the HULC gene could decrease RM susceptibility and protect patients against abortion. Meanwhile, it is shown that overexpression of HULC can enhance cells proliferation, invasion and migration without changing the mesenchymal stem cells typing and differentiation abilities [38]. Taken together, lncRNA HULC may also modulate RM susceptibility by altering the biological processes of cells. Yet further research is still required (Fig. 2).

#### CCAT2

CCAT2 (Colon cancer-associated transcript 2) is located on chromosome 8q24 with a 1752 bp lncRNA, which was initially identified in colorectal cancer [60, 61]. Bertucci et al. [39] indicated that the CCAT2 rs6983267 polymorphism is a risk factor for the development of inflammatory breast cancer. Meanwhile, studies have identified that the incidence of RM is associated with inflammation. Che et al. [40] compared the relationship between the polymorphisms of CCAT2 and the susceptibility to RM in 248 patients with RM and 392 healthy patients, and the result confirmed that the CCAT2 rs6983267 G allele is related to a reduced risk of RM. This demonstrates that the variant of rs6983267 G may serve an important role in minimizing the number of RM patients.

#### MALAT1

MALAT1 (Metastasis associated in lung adenocarcinoma transcript 1) is located on chromosome 11q13 with an 8.5 kb lncRNA, which was initially discovered in early-stage non-small cell lung cancer [62].

It has been confirmed that the MALAT1 expression level is reduced in villus samples of RM patients, and the regulation of MALAT1 is a contributing factor to RM pathogenesis, suggesting that the polymorphism of MALAT1 gene may be related to RM [41]. Furthermore, Che [42] et al. explored the association between MALAT1 gene polymorphism (rs619586) and the combined effects of RM susceptibility and protective genotypes according to age and number of miscarriages. The AG/GG variant is more protective in women under 35 years old and in women who have experienced 2 or 3 miscarriages, compared with the rs619586 AA variant [42]. However, the sample size of these studies was relatively small. Furthermore, larger research samples and additional experimental methods should be used to explore the specific role of lncRNAs in RM, which will be beneficial to determine the cause of RM.

### Endometrial receptivity

The endometrium is a layer of the inner wall of the uterus that changes periodically with changes in oestrogen and progesterone [63]. Cyclical changes in the endometrium provide it with a specific ability, endometrial receptivity, which is the ability of the endometrium to accept embryos [64]. In each menstrual cycle, embryos can only be accepted by the endometrium during the implantation window. After the fertilized egg reaches the endometrial cavity, it penetrates the epithelium of the endometrial surface through interactions with long mucin molecules (adherence phase) [65]. Then, the endometrial adhesion molecule αv/β3 integrin (β3) (adhesion phase) tightly adheres to the surface of the endometrium [66]. These biological processes provide adequate preparation for embryonic development and are indispensable for a normal pregnancy.

#### H19

H19 is located within chromosome 11p15.5 and is expressed in most cells, exclusively by the maternal allele [67]. It has been confirmed that the receptivity of the endometrium during the implantation window decreases when integrin β3 expression decreases [68]. Zeng et al. [43] found that Let-7, as a molecular sponge, is adsorbed by lncRNA H19 to regulate the transcription and translation of integrin β3 (ITGB3) (Table.3). Decreased expression of lncRNA H19 and integrin β3 can be detected in patients with RM, and this process is positively correlated with RM, which reduces endometrial receptivity [43].

**Table 3:** lncRNA expression, targets and effects on RM

lncRNA	Expression	Target	Effect	Refs
H19	Downregulated	miRNA let-7/ITGB3	Inhibits the adhesion and invasion of HTR-8 cell	[43]
Lnc-49a	Downregulated	CD49a	Inhibits the Migration, adhesion, and cytotoxic Activity of dNK cells	[46]
Lnc-SLC4A1-1	Upregulated	NF-κB/CXCL8	Induces immune responses in trophoblast cells	[47]
LncRNA SNHG7-1	Downregulated	miR-34a/WNT1	Inhibits proliferation and invasion of trophoblast cells	[48]
LncRNA HOTAIR	Downregulated	PIK3-AKT signalling pathway	Inhibits the migration and invasion of trophoblast cells	[49]
Lnc-HZ08	Upregulated	PI3K/p-AKT/P21/CDK2 pathway	Inhibits proliferation, migration, and invasion of trophoblast cells	[52]
Lnc-HZ01	Upregulated	MXD1/METTL14	Inhibits proliferation of trophoblast cells	[53]
H19	Downregulated	miR-675	Inhibits proliferation of trophoblast cells	[54]

*lncRNA* long non‑coding RNA, *ITGB3* inhibit integrin β3, *HOTAIR* HOX antisense intergenic RNA, *CXCL8* C-X-C motif ligand 8, *WNT1* Wnt Family Member 1, *METTL14* Methyltransferase-like 14

#### TUNAR

TUNAR (TCL1 Upstream Neural Differentiation-Associated RNA) is an approximately 1.0 kb lncRNA expressed explicitly in the human central nervous system and affects cell differentiation, proliferation, and apoptosis [69, 70]. A study indicated that endometrial biopsies of the late proliferative phase were collected from patients with or without recurrent implantation failure, and the results showed luteinizing hormone (LH) levels of + 2 and + 7 [44]. Meanwhile, the TUNAR expression level in endometrium was downregulated in patients with LH levels of + 7 and upregulated in RM patients. Multiple functions of TUNAR in endometrial epithelial cells (EECs) and endometrial stromal cells (ESCs) were investigated after transfection with pZW1-snoVector-TUNAR [44]. Wang et al. [44] showed for the first time that lncRNA TUNAR was expressed in the human endometrium and might be implicated in embryo implantation by regulating attachment of blastocyst to the endometrial epithelium and modulating the decidualization and proliferation of ESCs. Collectively, TUNAR may play a vital role in regulating endometrial receptivity.

#### Others

Feng et al. [45] collected 16 mid-luteal endometrial samples (including 8 from the experimental group of patients with RM and 8 from the control group with successful conception) and performed RT-PCR experiments, which demonstrated that the expression of lncRNA CECR3, ST7-OT3, DHRS4-AS1, C22orf34, RAMP2-AS1, and PNCT_HSA157732 were increased significantly in the endometrium of RM patients. Additionally, GO and KEGG pathway functional enrichment analyses confirmed that these six lncRNAs were associated with vascular proliferation, growth factor binding, immune activity, apoptosis, and synthesis of steroid hormones in the uterus to prepare the endometrium for embryo implantation [45]. The above studies indicated that lncRNAs could be used as predictive biomarkers of endometrial receptivity. However, the specific mechanism of lncRNAs remains a significant focus of research.

### The maternal-foetal interface

#### Cellular level

The maternal–fetal interface refers to the endometrium and extra terminal tissue during pregnancy, which is the area where the mother comes in direct contact with the fetus [71]. The maternal–fetal interface consists of decidual immune cells, decidual stromal cells, and trophoblasts [72].

Most immune cells in the decidua, which is crucial to pregnancy, belong to the NK family. NK cells in the decidua are different from killer cells in the traditional sense. Nonetheless, they are “trophoblast” cells that produce many cytokines without the central defensive toxicity of pbNK cells [73]. Although cytotoxic proteins are expressed in dNK cells, including granulysin, granzymes A and B, and perforin providing them cytolytic capacity, this cytotoxic machinery does not cause death of the invading trophoblast except potentially when responding to infection [74]. The cytotoxicity they display is reduced, which may be attributed to the mode of inhibition and activation of receptors expressed on the surface of dNK cells [75].

In addition, trophoblasts arise from the embryonic ectoderm, consisting of syncytiotrophoblasts and cytotrophoblasts. It is essential for pregnancy that trophoblasts invade the uterus. The formation of villous vessels in early pregnancy promotes the invasion of trophoblasts. If the formation of villous vessels is blocked, the invasion of trophoblasts will be obstructed, which will eventually lead to abortion. It has been shown that the migration and invasion of trophoblasts are associated with complex biochemical interactions, including increasing cell adhesion and enhancing cell proliferation [76].

##### Lnc-49a

Li et al. [46] examined the deciduae of 15 groups of patients with recurrent abortion and 15 groups of patients with normal abortion, which found that lnc-49a can positively regulate CD49a expression and maintain reduced cytotoxic activity. The adhesion and migration of dNK cells were downregulated, while the expression levels of interferon-γ granzyme B, and perforin in dNK cells were upregulated by a CD49a-neutralizing antibody which increased the killing ability of dNK cells [46]. It can be concluded that Lnc-49a can alter the homeostasis of the decidual microenvironment, leading to recurrent spontaneous abortion.

##### ANRIL

ANRIL (the long antisense non‐coding RNA at the INK4 locus) is located within chromosome 9p21, approximately 3.8 kb in length [77]. LncRNA ANRIL and mVEGF expression levels in villi of RM patients were positively correlated, and both were down-regulated, suggesting that lncRNA ANRIL may be down-regulated and may further inhibit villous vascular formation and trophoblast invasion in patients with RM by regulating the down-regulation of VEGF expression.

##### Lnc-SLC4A1-1

Lnc-SLC4A1-1 has been demonstrated to recruit NF-κB and bind to the CXCL8 promoter, which contributes to upregulation of CXCL8 [47]. Meanwhile, the elevation of CXCL8 intensifies the inflammatory response by inducing TNF-α and IL-1β, which can lead to the apoptosis of trophoblasts [47]. This finding represents a new step in determining the mechanism of recurrent spontaneous abortion.

##### SNHG7

SNHG7 (small nucleolar RNA host gene 7) is a known lncRNAs, with a total length of 2176 bp, which is located within chromosome 9q34 [78]. Research has indicated that abnormal expression of the Wnt/β-catenin pathway may be involved in RM. Xiang et al. [48] used qRT-PCR to determine that lncRNA SNHG7-1 was downregulated in HTR-8/SVneo cells. Knockdown of lncRNA SNHG7 can inhibit the proliferation, invasion and induce apoptosis in HTR-8/SVneo cells. When miR-34a was overexpressed in HTR-8/SVneo cells, WNT1 expression was significantly downregulated because miR-34a inhibits WNT1 expression. Hence, targeting lncRNA SNHG7 by miR-34a plays a vital role in the progression of RM through the Wnt/β-catenin signaling pathway, providing valuable therapeutic targets for patients with RM.

##### HOTAIR

HOTAIR (HOX transcript antisense RNA) is approximately 2.2 kb in length, which is located in chromosome 12q13 [79]. Zhang et al. [49] found that YY1, as a transcriptional activator of lncRNA HOTAIR, activates the PI3K-AKT signaling pathway to promote MMP-2 expression by enhancing the migration and invasion of trophoblasts. In another study, Wang et al. [50] separated primary villous trophoblasts to examine the P53-MALAT1 axis, showing that P53 expression was upregulated in the villi of RM patients and the lncRNA MALAT1 expression level was downregulated. Moreover, P53 is a negative regulator that has a vital role in many biological processes, including the cell cycle, apoptosis and differentiation [51]. Upregulation of P53 expression promotes cell apoptosis and reduces the survival rate of trophoblasts, leading to RM.

##### Lnc-HZ08

Increasing evidence has indicated that pregnant women might miscarry after exposure to environmental benzo(a)pyrene(BaP). Additionally, benzo(a)pyren-7,8-dihydrodiol-9,10-epoxide(BPDE), the ultimate metabolite of BaP, could induce dysfunction in trophoblasts. The expression of lnc-HZ08 was significantly upregulated in both RM tissue and BPDE-treated cells, and overexpression lnc-HZ08 suppressed the PI3K and AKT protein levels, and the downstream proteins CDK2 and p-P21 [52]. Meanwhile, lnc-HZ08 promoted the ubiquitin degradation of PI3K by promoting the interaction between CBL and PI3K in trophoblasts; therefore, lnc-HZ08 may negatively regulate proliferation, invasion and migration by inhibiting the PI3K/p-AKT/P21/CDK2 signaling pathway in BPDE-exposed trophoblasts [52].

##### Lnc-HZ01

In trophoblasts, lnc-HZ01 promotes the transcription of MXD1 mRNA by upregulating c-JUN, promotes stability of MXD1 by upregulating its deubiquitinating enzyme USP36, and eventually upregulates the level of MXD1 protein in trophoblasts nucleus [53]. However, MXD1 promotes the transcription of METTL14 mRNA and upregulates the level of lnc-HZ01 m6A RNA methylation, which promotes the stability of lnc-HZ01 and increases its expression level. Therefore, lnc-HZ01 and MXD1 upregulate each other, forming a positive self-feedback loop. Meanwhile, BPDE could activate this loop by upregulating the MXD1/METTL14/lnc-HZ01 and lnc-HZ01/MXD1 signaling pathways. Once the loop is activated by BPDE exposure, EIF4E expression levels are upregulated, and the proliferation of trophoblasts is suppressed, which eventually leads to miscarriage [53]. Therefore, lnc-HZ01 modulates both the proliferation of trophoblasts and the occurrence of miscarriage. Together, the above studies have shown that trophoblasts mainly control RM, and disrupting the function of trophoblasts may lead to RM.

#### Organizational level

The placenta is an organ that conducts material exchange between the mother and the foetus and is composed of the amniotic membrane, phyllodes chorionic membrane, and decidua basalis. When the fertilized egg is implanted, it adheres to the maternal endometrium and trophoblasts of the embryo invade the endometrium, which eventually results in placentation [72]. Furthermore, normal development of the placenta is vital for a normal pregnancy.

##### H19

lncRNA H19 is the template of miR-675. The stem-loop of miR-675 has been indicated to be one of the most conserved features of H19 RNA in the evolution of mammals. Examination of the embryonic tissues of 43 RM patients and 55 control patients demonstrated that the expression level of lncRNA H19 was upregulated in RM patients. Liu et al. [54] used H19-silenced JEG-3 cells that were transiently transfected with siRNA to detect the downregulation of miR-675 expression. When the miR-675 expression is downregulated, the growth of the placenta will also be inhibited. Further experiments confirmed that NOMO1 is a target gene that regulates H19/miR-675 in human trophoblasts [54]. Intriguingly, another studies have shown that lncRNA H19 slows placental growth in the second trimester of pregnancy by downregulating the RNA-binding protein HuR, which usually hinders miR-675 processing during the Drosha stage [55]. Increased expression of miR-675 in the placenta is often accompanied by downregulation of lgf1r, which can lead to slower growth, indicating that miR-675 is negatively correlated with placental development. Conclusions drawn from the role of lncRNA H19 in RM should not be ignored because they will provide a solid foundation for treatment.

## Future perspectives

In recent years, studies of lncRNAs have made breakthroughs, but due to the abundance of lncRNAs, these studies only provide a small amount of information, and many functions of lncRNAs have not been explored. In addition, the vast majority of the thousands of mammalian lncRNAs that have been identified thus far remain entirely uncharacterized. Therefore, the full mechanisms by which these molecules regulate biological processes remain unknown. LncRNAs have a wide range of functions and are involved in regulating the occurrence and development of many diseases, which is the main reason why they have become a focus of research in recent years. Existing experimental studies have a significant flaw because most lncRNA functions are explored at the cellular level, while in vivo animal experiments are rarely carried out. Designing animal experiments to explore the functions and mechanisms of lncRNAs is a problem that urgently needs to be solved. Experimental methods and tools for the study of lncRNAs will be developed in the future, which will provide a theoretical basis for the diagnosis and treatment of RM.

## Data Availability

Not applicable.

## Abbreviations

CCAT2: Colon cancer-associated transcript 2
CXCL8: C-X-C motif ligand 8
HOTAIR: HOX antisense intergenic RNA
HULC: Highly upregulated in liver cancer
ITGB3: Inhibit integrin β3
lncRNA: Long non‑coding RNA
MALAT1: Metastasis associated lung adenocarcinoma transcript-1
METTL14: Methyltransferase-like 14
RM: Recurrent miscarriage
TUNAR: TCL1 upstream neural differentiation-associated RNA
WNT1: Wnt family member 1
